# Developmental Tuning of Epigenetic Clock

**DOI:** 10.3389/fgene.2018.00584

**Published:** 2018-11-22

**Authors:** Alexander Vaiserman

**Affiliations:** Institute of Gerontology, Kiev, Ukraine

**Keywords:** DNA methylation, age-related disease, aging rate, developmental programming, epigenetic clock, epigenetic drift

## Abstract

Research in the field of gerontology has traditionally focused on later life stages. There is increasing evidence, however, that both the rate of age-related functional decline and the later-life health status can be programmed during early development. The central role of epigenetic mechanisms (methylation of DNA, histone modifications and regulation by non-coding RNAs) in mediating these long-term effects has been elucidated. Both rate and direction of age-associated change of epigenetic patterns (“epigenetic drift”) were shown to be largely dependent on early-life environmental conditions. Inter-individual divergences in epigenetic profiles may arise following the stochastic errors in maintaining epigenetic marks, but they may also be adaptively mediated by specific environmental cues. Recent cohort studies indicate that ticking rate of epigenetic clock, estimated by a DNA methylation-based methods, may be developmentally adjusted, and that individual’s discrepancies among epigenetic and chronological age would be likely programmed early in development. In this Perspective article, recent findings suggesting the importance of early-life determinants for life-course dynamics of epigenetic drift are summarized and discussed.

## Introduction

Genetic and lifestyle factors have been traditionally considered as main determinants of aging rate and longevity. However, accumulating data indicate that both individual’s aging trajectory and population mortality rate may substantially depend on developmental conditions (Tarry-Adkins and Ozanne, 2014; Vaiserman et al., 2018). The mechanistic pathways underlying such life-long effects are largely unknown, but modulation of epigenetic regulation of gene expression appears to be the most plausible explanation (Bianco-Miotto et al., 2017). Epigenetic modifications refer to heritable changes in gene expression occurring without changes in DNA sequence. Main components of epigenetic control include DNA methylation, histone modifications, and regulation by non-coding RNAs. Initially, it was assumed that stochastic errors in maintaining epigenetic marks (“epimutations”) occur during the life course due to limitations in epigenetic settings of maintenance and repair functions (Holliday, 1987). Since age-related accumulation of epimutations inevitably leads to impairment of the normal gene responsiveness to environmental stresses and to gradual loss of phenotypic plasticity, this process is commonly believed to be one of the main causes of aging (Gravina and Vijg, 2010). In addition, since age-related loss of DNA methylation may result in chromosome instability, and hypermethylation of DNA in promoter regions of tumor suppressor genes can cause their suppression, accumulation of epimutations with age could play an important role in the initiation and progression of different cancers (Jung and Pfeifer, 2015; Feinberg et al., 2016).

An accumulation of mistakes in maintaining normal epigenetic patterns leads to an increase in variability of epigenetic marks throughout the individual’s life course. This process currently referred to as “epigenetic drift” results in a gradual impairment of the body’s homeostatic mechanisms and appears to be a crucial hallmark of aging and an important determinant of longevity (Mendelsohn and Larrick, 2017). The earliest evidence for epigenetic drift in humans was provided from studies of monozygotic (MZ) twins. Within-pair comparison of such twins provides a useful model for investigation of factors regulating epigenetic variability by controlling for genetic variation. Despite MZ twins have the same genetic background their epigenetic profiles have been shown to gradually diverge with age (Fraga et al., 2005). Remarkably, most apparent divergences in epigenetic profiles have been observed in MZ twins who, according to questionnaire responses, had spent less of their lifetime together and/or had more differing natural health–medical history. Similarly, in recent study by Li et al. (2018), the correlation in genome-wide average DNA methylation levels between non-twin first-degree relatives was shown to increase with time living together and decrease with time living apart. An interaction between environmental conditions and age-related methylation divergence was also confirmed in a longitudinal, genome-scale analysis of DNA methylation in MZ twins from birth to 18 months (Martino et al., 2013). These findings collectively suggest that divergence in epigenetic profiles can occur not only via the stochastic epigenetic drift caused, e.g., by errors in the maintenance of DNA methylation patterns throughout DNA replication cycles, but may also be mediated directly by specific environmental cues (Cortessis et al., 2012; Cunliffe, 2015). From this, it is assumed that epigenome can respond to environmental challenges in an adaptive manner to maintain homeostasis and performance (Vaiserman, 2008, 2010; Herman et al., 2014). Thereby, the age-associated epigenome modification is increasingly seen as a process of memorization of environmental exposures experienced by organism over the lifetime. The epigenetic memorization likely affects the epigenetic drift rate. In this Perspective article, recent findings suggesting the importance of early-life determinants for life-course dynamics of epigenetic drift are summarized and discussed.

## Early-Life Determinants of Epigenetic Drift

Multiple lines of evidence indicate that both direction and rate of age-associated epigenetic drift largely depend on early-life environmental conditions (Li and Tollefsbol, 2016). This is not surprising, since it is well known that the epigenome (the whole-genome totality of epigenetic marks) is highly plastic during the early developmental period, especially throughout the establishment of the cell lineage-specific profiles of gene expression (Iurlaro et al., 2017; Burns et al., 2018). In mammals, including humans, windows of early-life epigenetic plasticity extend from preconception through weaning (Vaiserman, 2015). After the establishment throughout early developmental stages, most epigenetic marks are stably propagated in course of numerous cell divisions. Such epigenetic cellular memory allows to maintain stable profiles of gene expression in particular cell lineages throughout lifetime. This process is commonly referred to as “developmental epigenetic programming” (Hochberg et al., 2011). Importance of this period to future health status underlies the concept of the “first 1000 days,” prioritizing gestation and first 2 years after birth as a critical developmental period (Garmendia et al., 2014). According to the concept of predictive adaptive response, early-life cues can be used by an organism to rearrangement of the epigenome in a way that provides greatest fitness dividends in future life (Bateson et al., 2014). If resulting phenotype is properly matched to predicted environmental conditions, such adaptive strategy results in survival benefits in adult life. If adaptive epigenetic fine-tuning in early life is, however, incorrect and a mismatch exists between the further living conditions and the developmentally programmed phenotype, it may cause increased disease risk in adulthood (Godfrey et al., 2007). These conceptual considerations seem particularly relevant in the context of the topic discussed. The research findings suggesting the importance of early-life determinants for epigenetic programming of aging phenotypes and dynamics of epigenetic drift are presented in subsequent sections.

### Evidence From Animal Models

Since human data are scarce owing to restricted access to suitable biological materials, the most direct evidence for the role of epigenetic regulation in developmental programming of aging and longevity phenotypes came from animal models. In Merkwirth et al. (2016) study, perturbation of mitochondria throughout larval development not only delayed aging but also maintained unfolded protein response [UPR(mt)] signaling in *Caenorhabditis elegans*, assuming an epigenetic mechanism contributing to both lifespan and mitochondrial proteostasis across life course. In particular, reducing the function of lysine demethylases, JMJD-1.2/PHF8 and JMJD-3.1/JMJD3, has been shown to be able to potentially suppress longevity and UPR(mt) induction, while gain of function was sufficient to extend lifespan in a UPR(mt)-dependent manner. In the Greer et al. (2010) study, the ASH-2 trithorax complex, which trimethylates histone H3 at lysine 4 (H3K4), was identified as another important player in epigenetic programming of longevity in nematode. Remarkably, developmental activation of a particular longevity-regulating signaling pathways caused by misregulation of H3K4me2, led to a transgenerational lifespan extension in F2-F4 nematode offspring, potentially indicative of epigenetic memory (Greer et al., 2011). The possibility of such epigenetically mediated transgenerational effects on the worm’s longevity has been also confirmed in more recent studies (Alvares et al., 2014; Greer et al., 2016; Kishimoto et al., 2017).

Transgenerational effects on reproductive activity and longevity of *Drosophila melanogaster* induced by post-eclosion manipulation with dietary protein content were reported by Xia and de Belle (2016). In this research, both low- and high-protein diets reduced lifespan, while the intermediate-protein diet significantly extended longevity until F3 generation. In a subsequent study, feeding with a low-protein diet throughout the post-eclosion period resulted in a shortened life expectancy in F0 generation as well as in F2 offspring. These effects were accompanied by upregulating the H3K27-specific methyltransferase, E(z), and enhanced levels of H3K27 trimethylation, H3K27me3 (Xia et al., 2016). Interestingly, both RNAi-mediated knockdown of E(z) and pharmacological inhibiting its enzymatic function with a specific inhibitor of histone methyltransferase, Tazemetostat (EPZ-6438), lowered H3K27me3 levels across generations. In addition, Tazemetostat completely abolished the lifespan-shortening effect of the parental low-protein diet.

In rodent models, confirmatory evidence was also obtained for the role of factors such as xenobiotic exposure, stress, and malnutrition in developmental epigenetic programming of pathways contributed to the control of aging and longevity (for reviews, see Vaiserman, 2014; Tarry-Adkins and Ozanne, 2017; Ambeskovic et al., 2018). For example, in the study by Heo et al. (2016), prenatal malnutrition led to disrupted patterns of DNA methylation and dysregulation of transcriptional activity of genes implicated in aging-related processes and development of metabolic disorders in young (9-week-old) rat offspring. Remarkably, these dysregulated epigenetic patterns have been similar to those revealed in aged (20-month-old) offspring.

### Evidence From Twin Models

The evidence that epigenetic drift can be developmentally programmed was mostly obtained from studies in birth-weight discordant MZ twins. Birth weight was used in these studies as a proxy for prenatal conditions. Such research design generally relies on investigating twins raised in shared family environments, which may provide control not only for common genetic background but also for similar postnatal rearing environment. In the study by Gordon et al. (2011), strong evidence of gene expression discordance in MZ twins at birth was found in two different cell types. Among genes showing most different expression patterns within pairs, there were genes involved in stress response, metabolism, and cardiovascular function. In several recent studies, evidence was also obtained that early-life-induced epigenetic alterations may persist into adulthood. Although very similar genome-wide profiles of DNA methylation were found in saliva samples from discordant for birth weight adult female MZ twins (Souren et al., 2013), more recent studies revealed persistent differences in DNA methylation profiles. Importantly, most of these differentially methylated genes were known to be implicated in aging-associated processes. Pronounced differences in fetal growth (discordance in birth weight more than 20%) were significantly associated with DNA methylation changes in the *IGF1R* gene during adulthood (Tsai et al., 2015). In an epigenome-wide DNA methylation analysis conducted to examine adult MZ twins discordant for birth weight, a region on chromosome 1 has been identified as being differentially methylated for birth-weight discordance (Chen et al., 2016). This region covered two metabolism-associated genes, *CRYZ* and *TYW3*. Genome-wide methylome profiling in blood samples from adult MZ twins discordant for birth weight did not reveal any differences in DNA methylation patterns between twins, although particular sites displayed age-associated intra-pair differential methylation in highly birth weight-discordant twin pairs (Tan et al., 2014). The evidence that life-course dynamics of epigenetic drift is largely dependent from early-life programming events was also obtained from non-MZ twin models. In analyzing results from seven twin and/or family studies, Li et al. (2018) found that correlation in genome-wide average DNA methylation levels is very high at birth and remains high enough throughout life course in both twins and non-twin first-degree relatives. On the basis of these data, the authors concluded that genome-wide DNA methylation levels are determined *in utero* by prenatal environmental exposures, and these effects persist throughout life.

### Quasi-Experimental Evidence

Research findings indicative of contribution of epigenetic mechanisms to developmental programming of adverse later-life health outcomes were also obtained in quasi-experimental studies. Such research design (also referred to as “natural experiment”) is defined as “naturally occurring circumstances in which subsets of the population have different levels of exposure to a supposed causal factor, in a situation resembling an actual experiment where human subjects would be randomly allocated to groups” (Last, 1995). The Dutch famine, that took place in the German-occupied part of Netherlands in 1944–1945, is the most studied in this respect. The signs of accelerated aging were repeatedly reported in the cohort born during this famine, including impaired physical function, lowered cognitive performance, more atherogenic plasma lipid profile, enhanced stress responsivity, increased risk for depression and cardiometabolic disorders (Lumey et al., 2011; Roseboom et al., 2011; Bleker et al., 2016), and also the enhanced mortality rate (van Abeelen et al., 2012) at older ages. These adverse health outcomes were accompanied by persistent epigenetic changes. Whereas no association has been reported between prenatal exposure to the Dutch famine and the overall level of DNA methylation in adult life (Lumey et al., 2012), levels of DNA methylation of particular genes have been strongly associated with prenatal exposure to famine in the whole blood samples from adult offspring. Among the genes found to be differentially methylated between the famine-exposed subjects and non-exposed control individuals, there were genes known to be implicated in the development of cardiometabolic phenotypes, such as *IGF2* (Heijmans et al., 2008), and also *GNASAS*, *IL10*, *LEP*, *ABCA1*, *INSIGF*, and *MEG3* (Tobi et al., 2009). More recently, differential methylation of genomic regions extended along pathways associated with growth and metabolism was observed in persons periconceptionally exposed to famine (Tobi et al., 2014). Early, but not mid or late gestation, was identified as a critical time window for inducing persistent changes in DNA methylation profiles (Tobi et al., 2015). Even though it has not been reported until now whether it is a correlation between the DNA methylation and gene expression levels, the revealed DNA methylation modification was clearly related to impaired metabolic homeostasis in adult individuals who were prenatally exposed to famine (Lumey et al., 2007; Tobi et al., 2018). Similar results were obtained in studying long-term consequences of the perinatal exposure to famine in rural Bangladesh. In this study, those offspring who were exposed to famine perinatally were demonstrated to be at higher risk of developing obesity and type 2 diabetes during adulthood in comparison with unexposed control subjects. These health outcomes were associated with significant differences in methylation levels in metastable epialleles such as *PRDM-9*, *VTRNA2-1*, *PAX8*, near *BOLA*, near *ZFP57*, and *EXD3* (Finer et al., 2016).

## Developmental Adjustment of Epigenetic Clock

Recently, DNA methylation has gained significant interest as a powerful biomarker of aging allowing to quantify the individual aging rate and inter-individual variations in functional decline and timing of disease onset during the life course (Levine et al., 2018). In particular, the multi-tissue algorithm developed by Horvath (2013) allows to produce age estimates which correlate with chronological age well above *r* = 0.90 for full age range samples. Recent cohort studies indicated that ticking rate of epigenetic clock, estimated by DNA methylation-based methods, may be developmentally adjusted, and that individual’s discrepancies between epigenetic and chronological age may be programmed early in life. The supporting evidence for this came mainly from cohort studies. In most of these studies, the differences between actual chronological age and calculated DNA methylation age have been estimated using Horvath’s method (Horvath, 2013). For example, dependence of adult rate of epigenetic aging (age acceleration, AgeAccel) from childhood conditions was evaluated in the study by Simpkin et al. (2016). AgeAccel was defined as residual from regressing epigenetic age on actual age. The association between birth weight and AgeAccel during adolescence was found in two birth cohorts in this study. In analysis of the profiles of DNA methylation across five time-points in mother-child pairs from a United Kingdom birth cohort, adolescent AgeAccel was associated with maternal alcohol consumption during pregnancy. In a subsequent longitudinal study conducted with the same birth cohort, strong associations between AgeAccel and several developmental characteristics including height, weight, fat mass, and body mass index during childhood and adolescence have been demonstrated (Simpkin et al., 2017). An inverse association between pubertal tempo and AgeAccel in girls was shown in the longitudinal Growth and Obesity Cohort Study (Binder et al., 2018). Similar findings were obtained in two Finnish follow-up cohorts where AgeAccel levels were established in pre-adult life and remained unchanged during the rest of the lifetime, even in oldest-old ages (Kananen et al., 2016). On the basis of these findings, the epigenetic clock theory of aging was recently proposed by Horvath and Raj (2018), which postulated that epigenetic clock links developmental processes to aging.

## Hypothetical Considerations

**FIGURE 1:**
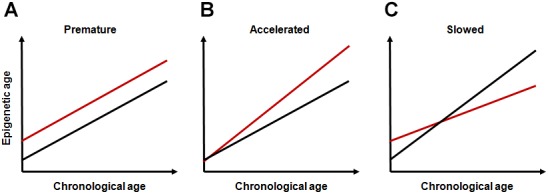
Hypothetical modes of life-course dynamics of epigenetic aging. **(A)** premature epigenetic aging; **(B)** accelerated epigenetic aging; and **(C)** slowed epigenetic aging. In all panels, red lines represent subjects exposed to adverse early-life events and black lines represent unexposed subjects.

Based on the conceptual frameworks and research findings above, it can be assumed that life-course dynamics of epigenetic aging can depend on early-life events and that the mode of this dependence can be different depending on the stage affected and on the type, duration and intensity of exposure. Most data on this relationship correspond to common intuitive notions. Adverse environmental exposures early in life can result in an elevated rate of accumulation of epimutations without accelerating the rate of epigenetic aging. This may increase intercept parameter without changing the slope parameter in the linear regression model describing this relationship (“premature epigenetic aging,” Figure 1A). Inappropriate developmental programming due to, e.g., mismatched epigenetic adaptation, may cause increase in slope (“accelerated epigenetic aging,” Figure 1B).

Some findings on long-term consequences of early-life programming events are, however, counterintuitive and require additional assumptions. For example, in analyzing the deceleration of the old-age mortality rate observed across developed countries over the second part of the XXth century, Yashin et al. (2001) suggested that some centenarians paradoxically come from an initially frail part of the cohort. The authors hypothesized that more vulnerable (and likely more labile) organism can better improve its stress reactivity than more robust (and rigid) one. This can result in survival advantages to originally more vulnerable organisms at older ages. The evidence for this assumption also comes from studies conducted to test hygiene hypothesis proposed by Strachan (1989) to explain the link between the lack of microbial exposure due to overhygienic conditions in childhood and high risk of allergic and autoimmune disorders in later life. The overhygienization is becoming increasingly widespread in modern world, and it is believed to cause increased antibiotic resistance and permanently increasing number of persons with weakened immunity in developed societies (Bloomfield et al., 2016). Although lack of microbial exposure may not be the only causal factor, the assumptions of this hypothesis have been repeatedly confirmed through both observational and experimental studies (Maia Rda and Wünsch Filho, 2013). Under-using of immune system early in life can likely cause not only atopic conditions, but also autoimmune states (Kramer et al., 2013). The latter seems especially important since immunosenescence are regarded now as one of the leading processes underlying aging. Importantly, although inflamm-aging is traditionally regarded as leading cause of most age-related disorders, such strictly negative interpretation of immunosenescence is challenged now by many immune*-*gerontologists. Age-associated immune changes are increasingly considered as adaptive or remodeling rather than solely detrimental (Fulop et al., 2018). Despite the fact that these immune changes can obviously cause various pathological conditions, these alterations may potentially contribute to developing extended longevity phenotypes. It is even suggested by the authors that without the presence of the immunosenescence/inflamm-aging, human longevity would be substantially shortened (Fulop et al., 2018). An important point in the context of the topic discussed is that complex interactions between immune and epigenetic pathways likely play an important role in these effects (Grolleau-Julius et al., 2010; Jasiulionis, 2018). Given the fact that these processes can have a significant impact on epigenome, they may likely decelerate the epigenetic clock-ticking rate by increasing the intercept parameter and by decreasing slope value (“slowed epigenetic aging,” Figure 1C), even though initial level of accumulation of epimutations could be higher in early-life exposed population than in unexposed one. Interestingly, in the study by Marioni et al. (2018), epigenetic age was shown to be increasing at a slower rate than chronological age across life course, especially in the oldest population. The selection bias in which healthier individuals are more likely to reach older ages was proposed to explain these results by the authors. The individual induction due to epigenetic adaptation might, however, be an alternative explanation. For a definitive conclusion, nevertheless, more thorough research is required.

## Conclusive Remarks

Research in the field of gerontology has traditionally focused on later stages of the life cycle. There is, however, increasing evidence that the rate of age-related functional decline and risk for aging-associated diseases can largely depend on developmental conditions. Growing awareness of the of developmental programming in the pathogenesis of adult-life chronic pathological conditions and unraveling the mechanisms involved led to rising interest in research in this area. The developmental programming processes would likely be of particular importance in modern developed societies due to significant lifestyle changes (westernized diet, sedentary behavior, etc.), which can often conflict with adaptive epigenetic strategies programmed during development.

Two important points follow from these considerations for future research and practical applications. First, further development of epigenetic methodology will provide an opportunity to identify individuals at risk for developing certain age-related pathological condition due to early-life malprogramming long before the clinical manifestation of the disease. Second, since epimutations, unlike genetic mutations, are potentially reversible, they may be corrected by specific nutritional and/or pharmacological interventions. The implementation of such epigenome-targeted interventions would allow to influence life-course dynamics of epigenetic age and slow down the ticking rate of the epigenetic clock to delay or decelerate the aging-related processes.

## Author Contributions

The author confirms being the sole contributor of this work and has approved it for publication.

## Conflict of Interest Statement

The author declares that the research was conducted in the absence of any commercial or financial relationships that could be construed as a potential conflict of interest.
